# Assessment of lumbopelvic–hip complex instability and segmental sequencing amongst softball athletes

**DOI:** 10.1080/23335432.2018.1481456

**Published:** 2018-07-24

**Authors:** Gabrielle Gilmer, Jessica Washington, Gretchen Oliver

**Affiliations:** School of Kinesiology, Auburn University, Auburn, AL, USA

**Keywords:** Single leg squat, kinetic chain, throwing

## Abstract

The purpose of this study was to examine the effects of lumbopelvic–hip complex (LPHC) instability on segmental sequencing and the maximum velocities during the overhead throw. Fifty softball athletes (164.0 ± 104.0 cm, 65.6 ± 11.3 kg, 16.3 ± 3.8 years) classified as either college, high school or youth performed three 60 ft overhead throws then executed bilateral single leg squats (SLS). Kinematics were recorded using an electromagnetic tracking system. Participants were classified as ‘unstable’ if they displayed knee valgus greater than 15° at 45° knee flexion in the descending phase of the SLS. One-way ANOVAs and Bonferonni post-hoc tests revealed no significant differences between stability groups in segmental sequencing and maximum velocities amongst the college, high school and youth participation level. When all athletes were grouped together regardless of age, there were still no significant differences observed between groups. These findings imply that segmental sequencing and maximum velocities are not a function of LPHC stability amongst this specific group of athletes. Additionally, the SLS may not accurately quantify LPHC stability in regards to throwing. The authors recommend that future studies repeat these methods amongst different athletic populations and continue to evaluate different clinical tests for LPHC stability.

## Introduction

When performing dynamic activities such as overhead throwing, the body acts as a kinetic chain comprising independent links connected in series (Putnam 1993). The throwing motion requires the kinetic chain to work in a sequential and coordinated manner to properly transfer energy from proximal to distal segments in attempt to reduce injury susceptibility (Putnam 1993). While the importance of the upper extremity (distal segments of the kinetic chain) in the throwing motion is well defined (Wilk et al. 2000; Fleisig et al. 2011; Keeley et al. 2012b; Wicke et al. 2013; Plummer and Oliver 2013a; Tanaka et al. 2016), the effects of the lower extremity (proximal segments of the kinetic chain) on the throwing motion are not well described.

The lumbopelvic–hip complex (LPHC) is the key component connecting the upper and lower extremities and serves as a direct link in the transfer of energy along the kinetic chain (Kibler et al. 2006). The LPHC comprises musculature that connects the abdomen, proximal lower extremity, pelvis, trunk and spine (Hodges and Richardson 1997). LPHC stability is defined as the ability to maintain postural control; specifically, this means maintaining control of the pelvis over the stride leg and the trunk over the pelvis during overhead throwing (Wilson et al. 2005; Kibler et al. 2006).

While the throwing literature has focused on the upper extremity, a relatively small proportion of the forces and loads experienced during an overhead throw are actually generated by the upper extremity (Kibler and Sciascia 2016). Efficient energy transfer is generated from the lower extremity, through the LPHC, to the upper extremity, and out to the wrist and hand for ball release (McMullen and Uhl 2000). Previous studies have reported that baseball pitching is successfully executed when angular momentum is conserved from the LPHC, thoracic spine and shoulder (Hirashima et al. 2002, 2008; Kageyama et al. 2015). Additionally, when LPHC instability results in altered energy transfer to the upper extremity, the elbow is at greater risk of injury in baseball athletes (Garrison et al. 2013; Chaudhari et al. 2014), while the forearm has been reported to be at risk in softball athletes (Bogenschutz et al. 2011).

The summation of speeds principle can be used to track the energy transferred through the body via segmental sequencing and states that the total energy in the kinetic chain is the sum of each segment’s individual energy contribution (Putnam 1993). Interruptions and losses of energy along the kinetic chain can ultimately result in a loss of segmental speed. Many different interruptions from the lower extremity, such as tight hamstrings, weak hip flexors, unstable LPHC and back injury, have been reported to alter the kinetic chain (Endo et al. 2014; Kibler and Sciascia 2016).

Clinically, the single leg squat (SLS) has become an assessment tool for LPHC stability and is shown to be a consistent indicator of lower extremity instability and potential injury (Graci et al. 2012). The SLS accentuates lower extremity weaknesses among athletes with unstable gluteal musculature, often resulting in relatively high knee valgus and external rotation, as well as increased hip adduction and internal rotation when compared to stable athletes (Graci et al. 2012). The SLS has been reported as a highly specific and valid clinical test for evaluating injury susceptibility (DiMattia et al. 2005; Kibler et al. 2006; Hollman et al. 2014). Although the SLS examination is validated, the exact value of knee valgus that an athlete becomes at risk has been debated and ranges from 2 to23° knee valgus (Zeller et al. 2003; DiMattia et al. 2005; Claiborne et al. 2006; Hollman et al. 2014; Yamazaki et al. 2019).

Therefore, the purpose of this study was to assess how segmental sequencing and maximum velocities of the pelvis, trunk, humerus and forearm during overhead throws differ as a function of LPHC stability. LPHC stability was evaluated using the SLS because the SLS has been validated as a clinical test that can accurately determine LPHC stability (DiMattia et al. 2005; Hollman et al. 2014). It was hypothesized that participants with any LPHC instability would display improper segmental sequencing and decreased maximum segmental velocities.

## Methods

### Experimental approach to the problem

The aim of this study was to examine the effects of LPHC instability on segmental sequencing and maximum segmental velocities in overhead throws. LPHC instability was determined using a SLS assessment, as this assessment is known to be a consistent test for lower extremity and LPHC instability (DiMattia et al. 2005; Graci et al. 2012; Hollman et al. 2014). Previous studies have shown that knee valgus in the SLS provides insight into weaknesses present in an athlete’s LPHC. The independent variable in this study was the stability group (Zeller et al. 2003; DiMattia et al. 2005; Claiborne et al. 2006; Bolgla et al. 2008; Crossley et al. 2011; Nakagawa et al. 2012; Yamazaki et al. 2019). The dependent variables were the segmental sequencing and maximum velocities of the pelvis, trunk, humerus and forearm.

### Participants

A convenience sample of 50 softball athletes (164.0 ± 104.0 cm, 65.6 ± 11.3 kg, 16.3 ± 3.8 years, 8.61 ± 3.62 years experience), regardless of playing position, were recruited to participate: 18 National Collegiate Athletic Association Division I collegiate players (165.0 ± 14.0 cm, 69.0 ± 8.0 kg, 20.9 ± 1.8 years, 13.06 ± 2.05 years experience), 17 high school players (167.0 ± 73.0 cm, 72.8 ± 8.9 kg, 15.5 ± 0.9 years, 7.24 ± 1.56 years experience) and 15 youth players (160.0 ± 87.0 cm, 55.0 ± 9.4 kg, 12.4 ± 0.7 years, 5.53 ± 1.36 years experience). As a softball athlete, these participants routinely threw overhead positional throws of 60 ft (18.3 m). Participants reported for testing prior to any throwing or vigorous activity on that day. Selection criteria included being medically cleared to participate in softball activities as well as having no previous lower or upper extremity injuries within the past 6 months. There were no exclusion criteria in place based on playing position (pitcher, catcher, utility, infield or outfield). The Institutional Review Board of Auburn University approved all testing protocols. Prior to data collection, all testing procedures were explained to each participant, and informed consent or parental assent was obtained.

### Procedures

**Figure 1.**
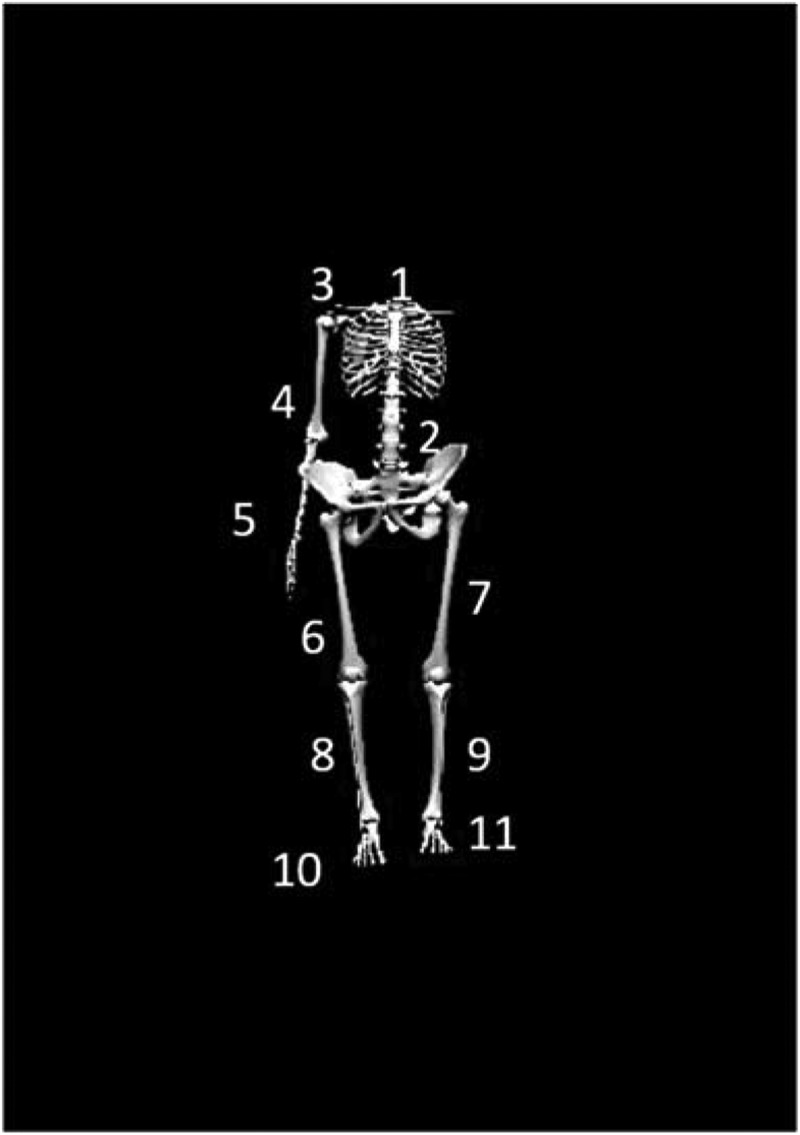
Electromagnetic sensors were placed at the following locations: (1) posterior aspect of the trunk at the first thoracic vertebrae (T1) spinous process; (2) posterior aspect of the pelvis at the first sacral vertebrae (S1); (3) flat, broad portion of the acromion on the throwing scapula; (4) lateral aspect of the throwing upper arm at the deltoid tuberosity; (5) posterior aspect of the distal throwing forearm, centered between the radial and ulnar styloid processes; (6 and 7) lateral aspect of the bilateral thigh, centered between the greater trochanter and the lateral condyle of the knee; (8 and 9) lateral aspect of the bilateral shank, centered between the head of the fibula and lateral malleolus; (10 and 11) and the bilateral dorsal aspect of the feet

Kinematic data were collected at 100 Hz using an electromagnetic tracking system (trakSTAR^TM^, Ascension Technologies, Inc., Burlington, VT, USA) synced with The MotionMonitor® (Innovative Sports Training, Chicago, IL., USA). Eleven electromagnetic sensors were affixed to the skin at the following locations (Figure 1): (1) posterior aspect of the trunk at the first thoracic vertebrae (T1) spinous process; (2) posterior aspect of the pelvis at the first sacral vertebrae (S1); (3) flat, broad portion of the acromion on the throwing scapula; (4) lateral aspect of the throwing upper arm at the deltoid tuberosity; (5) posterior aspect of the distal throwing forearm, centered between the radial and ulnar styloid processes; (6 and 7) lateral aspect of the bilateral thigh, centered between the greater trochanter and the lateral condyle of the knee; (8 and 9) lateral aspect of the bilateral shank, centered between the head of the fibula and lateral malleolus; (10 and 11) and the bilateral dorsal aspect of the feet (Keeley et al. 2012a; Plummer and Oliver 2013a, 2017; Oliver et al. 2017; Plummer et al. 2018). The T1 spinous process was identified through palpation of the seventh cervical process via flexion and extension, while the S1 spinous process was identified through the palpation of the left and right posterior superior iliac spine (Ernst et al. 2013). A twelfth, moveable sensor was attached to a plastic stylus for the digitization of bony landmarks (Wu et al. 2002, 2005; Oliver and Keeley 2010a, 2010b). In order to ensure accurate identification and palpation of bony landmarks, the participant stood in anatomical neutral throughout the digitization process. Using the digitized joint centers for the ankles, knees, hips, T12-L1 and C7-T1, a link segment model was developed.

Joint centers were determined by digitizing the medial and lateral aspect of a joint then calculating the midpoint between those two points (Wu et al. 2002; Oliver and Keeley 2010a, 2010b). The ankle and knee joints were defined as the midpoint between the digitized medial and lateral malleoli and medial and lateral femoral condyles, respectively, whereas the spinal column was defined as the digitized space between C7-T1 and T12-L1. A rotation method, validated as capable of providing accurate positional data (Veeger 2000; Huang et al. 2010), was utilized to estimate the joint centers of the shoulder and hips. The shoulder joint center was calculated from the rotation of the humerus relative to the scapula, while the hip-joint centers were calculated from the rotation of the femur relative to the sacrum. The rotation method consisted of the investigator stabilizing the joint then passively moving the limb into six different positions in a small, circular pattern (Huang et al. 2010).

Raw data regarding sensor position and orientation were transformed to locally based coordinate systems for each of the representative body segments. For the world axis, the *y*-axis represented the vertical direction. The positive *x*-axis was defined anterior to the *y*-axis and in the direction of movement. The positive *z*-axis was defined as orthogonal to *x* and to the right of *y*. Position and orientation of the body segments were obtained using Euler angle sequences that were consistent with the International Society of Biomechanics standards and joint conventions (Wu et al. 2002). More specifically, *ZX*′*Y*″ sequence was used to describe pelvis and trunk motion and *YX*′*Y*″ sequence was used to describe shoulder motion. All raw data were independently filtered along each global axis using a 4th order Butterworth filter with a cutoff frequency of 13.4 Hz (Oliver and Keeley 2010b; Wicke et al. 2013; Plummer and Oliver 2013a).

A 40 cm × 60 cm Bertec force plate (Bertec Corp., Columbus, OH, USA) was built into the surface from which all throws were made, such that the participant’s stride foot would land on the force plate during the throwing motion. Force plate data were only used to event mark the instance of stride foot contact during the throwing motion and were sampled at a rate of 1000 Hz.

Field distortion associated with electromagnetic tracking systems was previously reported to cause errors greater than 5° at a distance of 2 m from the extended range transmitter, but instrument sensitivity increases have reduced this error from near 10° prior to system calibration to 2° following calibration (Meskers et al. 1999; Day et al. 2000; Perie et al. 2002). The system was calibrated using previously established protocols prior to the collection of any data (Day et al. 2000; Oliver and Keeley 2010b; Plummer and Oliver 2013b). After calibration, the error in determining position and orientation of the electromagnetic sensors was less than 0.01 m and 2°, respectively. Intra-rater reliability of digitization was determined during a pilot study of nine collegiate softball athletes. The investigator reported an intra-rater reliability using the technique described above, of an ICC(3,*k*) of 0.75–0.93 for all measurements.

After sensor attachment and digitization, each participant was allotted an unlimited amount of time to warm-up (average warm-up time: 5 min) for acclimation to all testing procedures. Participants performed their own pregame warm-up because the investigators wanted participants to simulate what they would normally do before a game. The testing began only when the participant was self-declared ready to partake in the throwing protocol. Each participant executed three maximal effort overhead throws to a teammate who was 60 ft away with one foot on the base. An accurate, saved trial was one in which the participant threw the ball to their teammate allowing the teammate to catch the ball in the air without removing their foot from the base. Because these data were collected during the off-season, we chose to only collect three trials to eliminate the potential for fatigue. After completion of all throwing trials, the participant performed an SLS on each leg (Plummer et al. 2018). Participants were instructed to cross their arms over their chest, flex the non-testing leg at the knee to 90 , placing the lower leg behind the body, then squat as low as they could while maintaining balance and ascended to a neutral stance. Participants were allowed to practice SLS before data were recorded (average practice time 1 min).

Although there are many techniques outlined in the literature for measuring LPHC stability, no standard functional test has been accepted for clinical use. Thus, participants were classified as ‘unstable’ if they displayed knee valgus greater than 15° at 45° knee flexion in the descending phase of the squat on one leg or both legs (Zeller et al. 2003; DiMattia et al. 2005; Claiborne et al. 2006; Bolgla et al. 2008; Crossley et al. 2011; Nakagawa et al. 2012; Yamazaki et al. 2019). Knee valgus during the SLS were recorded using the same electromagnetic tracking system used to record throwing data. Previous studies have also demonstrated that trunk position is a valid indicator of instability in the SLS as well (Plummer et al. 2018). The authors chose to focus on knee valgus in an attempt to reveal insights mainly pertaining to the lower extremity and LPHC. Demographic information for all stability group amongst each age group is shown in Table 1.

**Table 1.** Demographics both stability group within each age group

Age group	Parameter	Stable	Unstable
College			
	Height (cm)	164.7 ± 18.8	165.3 ± 10.4
	Weight (kg)	73.3 ± 8.8	66.7 ± 6.5
	Age (years)	21.5 ± 1.4	20.6 ± 1.9
	No. of participants	6	12
High school			
	Height (cm)	171.3 ± 3.5	167.7 ± 8.1
	Weight (kg)	74.8 ± 6.8	75.1 ± 8.8
	Age (years)	15.8 ± 0.8	15.6 ± 1.0
	No. of participants	5	12
Youth			
	Height (cm)	162.9 ± 1.4	159.2 ± 9.3
	Weight (kg)	67.6 ± 2.4	53.0 ± 8.4
	Age (years)	12.5 ± 0.7	12.4 ± 0.8
	No. of participants	2	13
Overall			
	Height (cm)	164.3 ± 12.5	166.4 ± 13.5
	Weight (kg)	70.2 ± 7.8	72.5 ± 8.4
	Age (years)	17.7 ± 3.9	17.4 ± 3.3
	No. of participants	13	37

### Statistical analysis

**Figure 2.**
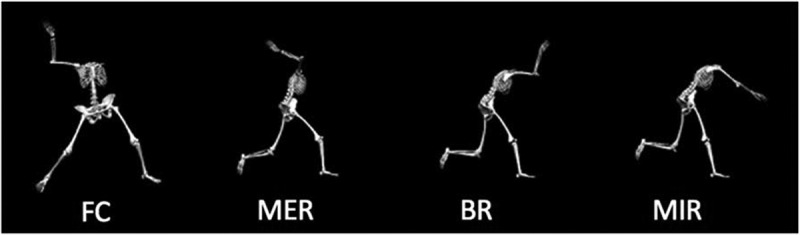
All throws were analyzed across four throwing events: foot contact (FC), maximum external shoulder rotation (MER), ball release (BR) and maximum internal shoulder rotation (MIR)

Kinematic data were averaged across the three trials of the 60 ft overhead throws and used for analysis. The throwing motion was defined by four events: (1) foot contact, (2) maximal shoulder external rotation, (3) ball release, and (4) maximal shoulder internal rotation (Figure 2). All data were processed using a customized MATLAB (MATLAB R2010a, MathWorks, Natick, MA, USA) script. Statistical analyses were performed using IBM SPSS Statistics 22 software (IBM Corp., Armonk, NY) for both normally and non-normally distributed data with an alpha level set a priori at *α* = 0.05.

Prior to analysis, Sharpio–Wilk tests of Normality were run. Results showed normal distributions for all data. All kinematic variables were analyzed using one-way ANOVAs (4 events × 2 groups) followed by Bonferroni post-hoc tests for all statistically significant findings. One-way ANOVAs were employed to examine differences in the maximum values of pelvis, trunk, humerus and forearm segmental velocities and segmental sequencing between the LPHC stability groups. The investigators chose not to examine the main effects of event of the segmental velocities, as these findings would only indicate that the participant’s body is changing speed as they throw the ball.

## Results

**Figure 3.**
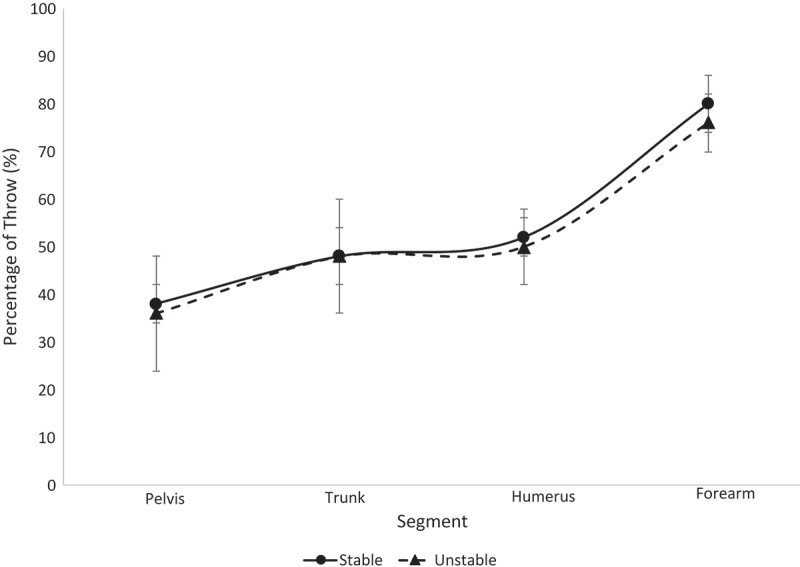
Segmental sequencing for each stability group within the college athletes is shown. No significant differences were observed

**Figure 4.**
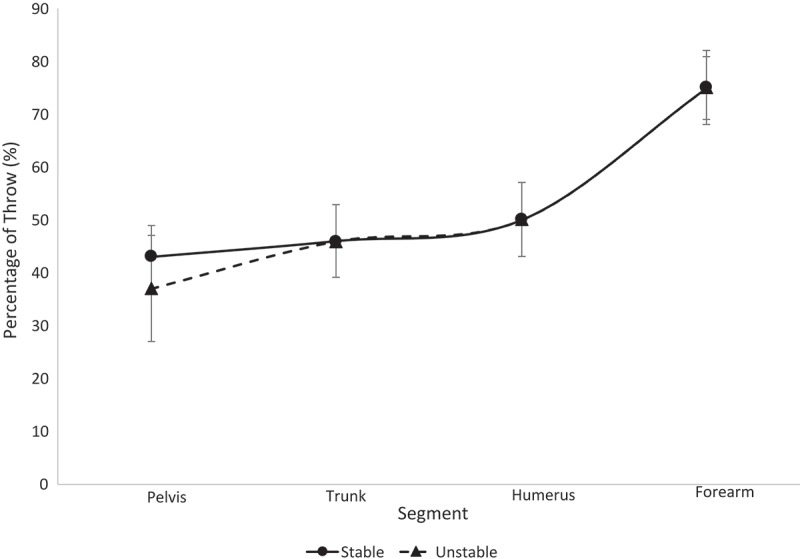
Segmental sequencing for each stability group within the high school athletes is shown. No significant differences were observed

**Figure 5.**
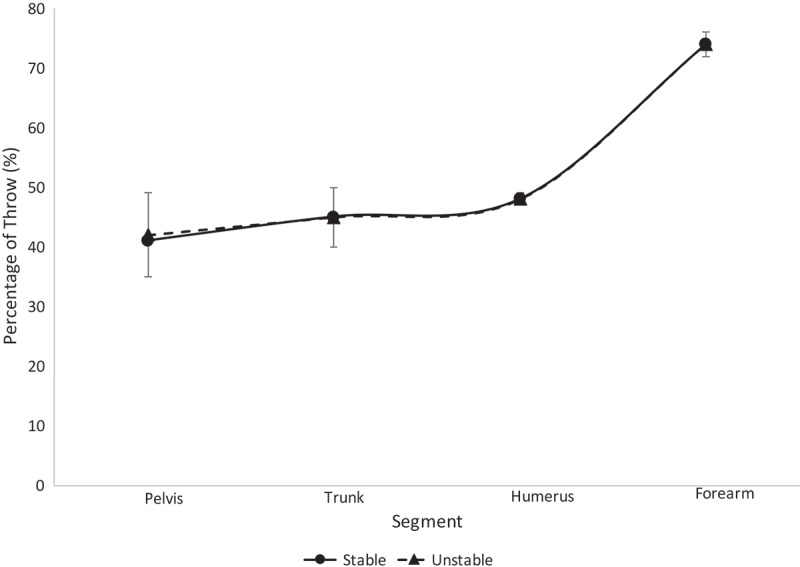
Segmental sequencing for each stability group within the youth athletes is shown. No significant differences were observed

Mean and standard deviations for the maximum velocities for the college, high school and youth level participants are shown in Tables 2–4, respectively. One-way ANOVAs revealed no significant differences (*p* > 0.05) in maximum angular and linear velocities between the stable and unstable groups for the college, high school and youth level participants. Means and standard deviations of the segmental sequencing for the college, high school and youth level participants are shown on Figures 3–5, respectively. One-way ANOVAs revealed no significant differences (*p* > 0.05) in segmental sequencing between the unstable and stable groups for the college, high school and youth level participants. A power analysis for the college, high school and youth athletes revealed corresponding statistical powers of 0.12, 0.10 and 0.09, respectively.

**Table 2.** The maximum values for each segment amongst college athletes within each stability group are shown

	(°/s)		m/s	
	Pelvis	Trunk	Humerus	Forearm
Stable	672 ± 58	876 ± 52	9.3 ± 0.8	13.2 ± 1.4
Unstable	662 ± 122	843 ± 98	9.2 ± 1.1	12.3 ± 1.0

No significant differences were found.

**Figure 6.**
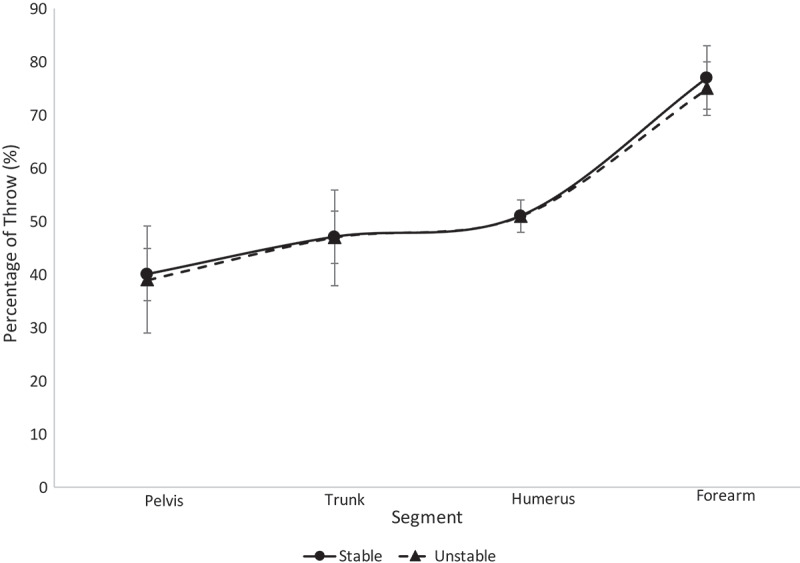
Segmental sequencing for each stability group within the overall athletic population is shown. No significant differences were observed

Mean and standard deviations for the maximum angular and linear velocities of the overall sample are shown in Table 5. The overall sample included a comparison between the stable and unstable groups and included all participants regardless of age. One-way ANOVAs revealed no significant differences in maximum angular and linear velocities. Means and standard deviations of the segmental sequencing are shown in Figure 6. One-way ANOVAs revealed no significant differences (*p* > 0.05) in segmental sequencing between the stable and unstable groups. A power analysis revealed a statistical power of 0.83.

**Table 3.** The maximum values for each segment amongst high school athletes within each stability group are shown

	(°/s)		m/s	
	Pelvis	Trunk	Humerus	Forearm
Stable	678 ± 112	850 ± 84	8.6 ± 1.0	11.2 ± 1.1
Unstable	640 ± 108	802 ± 90	8.7 ± 0.9	11.5 ± 1.2

No significant differences were found.

**Table 4.** The maximum values for each segment amongst youth athletes within each stability group are shown

	(°/s)		m/s	
	Pelvis	Trunk	Humerus	Forearm
Stable	698 ± 32	841 ± 89	8.7 ± 0.4	11.4 ± 0.0
Unstable	617 ± 96	762 ± 70	7.8 ± 1.0	10.7 ± 1.6

No significant differences were found.

**Table 5.** The maximum values for each segment amongst all athletes within each stability group are shown

	(°/s)		m/s	
	Pelvis	Trunk	Humerus	Forearm
Stable	678 ± 76	860 ± 66	9.0 ± 0.9	12.2 ± 1.4
Unstable	639 ± 108	801 ± 90	8.6 ± 1.1	11.5 ± 1.4

No significant differences were found.

## Discussion

The purpose of this study was to assess how LPHC stability, classified via knee valgus during the SLS assessment, affects segmental sequencing and the maximum segmental velocities of the pelvis, trunk, humerus and forearm among varying levels of softball athletes during 60 ft overhead throws. It was hypothesized that participants with any LPHC instability would display significantly altered maximums and segmental sequencing.

The results from this study reject the hypothesis. Across all ages and all stability groups, proper segmental sequencing was observed. This conclusion can be drawn based on the fact that the percentage of throw in which the maximum value occurred was sequential (i.e. the pelvis reached maximum angular velocity, then the trunk reached maximum angular velocity, etc.) (Chalmers et al. 2017; Sgroi et al. 2015). This finding suggests that segmental sequencing was not a function of LPHC stability in this group of softball athletes. All athletes displayed minimal separation of trunk and humerus sequencing, as seen in Figures 3–6, relative to the separation observed between other segments along the kinetic chain. The college athletes in both stability groups displayed a greater timing difference between the maximum trunk angular velocity and pelvis angular velocity than the high school and youth athletes. This finding was surprising to the authors, as it was expected that the LPHC unstable athletes would have similar pelvis and trunk maximum angular velocity timing, while stable athletes would have a notable difference in the timing of their maximum pelvis and trunk angular velocities. Previous studies have found that athletes who are unstable are not as efficient in utilizing their entire kinetic chain, resulting in lower ball speeds and segmental angular velocities (Kibler et al. 2006; Saeterbakken et al. 2011; Kageyama et al. 2015). Since there were no differences in segmental sequencing across groups, the authors speculate that compensations are being made amongst the unstable group in order to maintain the integrity of the kinetic chain.

The youth and high school participants displayed similar sequencing for the trunk, humerus and forearm in both stability groups. These findings were interesting as the authors expected the high school participants would have greater postural control than the youth participants due to their stage of development, since most female athletes do not start strength and condition training until they are in high school. When all athletes were grouped together (overall), the stability groups displayed the exact same sequencing of the trunk and humerus. Though we were able to separate the three levels of participants and overall athletic population into stable and unstable groups based on their SLS performance, it is possible that both stability groups were inefficient in LPHC control during the dynamic movement of throwing. While the SLS has been used as an assessment for LPHC control in previous studies, it may be necessary for sports medicine staff to use more dynamic assessments, such as the drop vertical jump or single-leg crossover drop down, to appropriately assess LPHC stability in dynamic activities such as throwing. In addition, future research should consider looking into single-leg positions that exist within the throwing motion itself to evaluate risk for injury (i.e. during throwing, athletes are on single-leg support for the majority of the motion, thus insight into injury prone mechanics could prove valuable).

Although not significantly higher, amongst the college athletes, the stable athletes displayed larger maximum velocities across all segments. Generally, across both stability groups and all age groups, the maximum angular velocity of the trunk was greater than the maximum angular velocity of the pelvis, and the maximum linear velocity of the forearm was greater than the maximum linear velocity of the humerus. This finding reiterates the importance of LPHC stability in energy transfer from the lower to upper extremity during dynamic throwing movement (Saeterbakken et al. 2011; Kibler et al. 2013; Chu et al. 2016; Naito et al. 2017). Because both groups were seemingly capable of properly transferring energy from segment to segment, it is likely that the unstable group could possibly be compensating in order to achieve proper sequencing. These findings again imply that segmental sequencing was not a function of LPHC stability.

Limitations of this study include small numbers of varying populations. Within the age-specific comparisons, each stability group had no more than 12 participants. While the overall comparison did have a larger sample size, the varying ages and stages of development amongst the participants may have affected results. In addition, all participants from this study are from the Auburn/Opelika, Alabama area. The implication of these results in other regions should be done with caution.

## Conclusion

Efficient and effective utilization of mobility and stability along the kinetic chain is crucial in the overhead throw. The findings of the current study suggest that LPHC stability, as evident by knee valgus during the SLS, does not influence segmental sequencing or the maximum segmental velocities of the pelvis, trunk, humerus and forearm during an overhead throw. Most injuries in throwing are a result of overuse in nature, meaning that the three trials in this study may not have quantified enough of what was actually going on with these athletes, since data were collected while the athlete was well rested. For more accurate quantification of LPHC stability and to truly observe its effects, longer bouts of throwing may be necessary for appropriate evaluation. Future studies should consider simulated game designs to further observe the effects of LPHC stability on longer time periods.

The authors recommend that future studies continue to assess different dynamic and easily applicable methods for classifying LPHC instability. This study not only resulted in no significant differences but also several of the average values between groups were exactly the same. The similarities between segmental sequencing and maximum angular velocities lead the authors to believe that more rigorous assessments, such as the drop vertical jump, may be necessary to properly evaluate LPHC stability’s effects on segmental sequencing and maximum angular velocities.

In addition, the lack of findings from the current study may suggest that multiple variables should be considered when using clinical tests when determining LPHC instability. As mentioned previously, the authors chose to use knee valgus as the instability indicator because of the focus on the lower extremity and LPHC. Previous studies have used trunk position (Plummer et al. 2018). There is a trade-off between complexity and accuracy when introducing multi-variable classification systems, but the authors believe this is worth investigating in the future.
